# Physiological characteristics of *Magnetospirillum gryphiswaldense* MSR-1 that control cell growth under high-iron and low-oxygen conditions

**DOI:** 10.1038/s41598-017-03012-4

**Published:** 2017-06-05

**Authors:** Qing Wang, Xu Wang, Weijia Zhang, Xianyu Li, Yuan Zhou, Dan Li, Yinjia Wang, Jiesheng Tian, Wei Jiang, Ziding Zhang, Youliang Peng, Lei Wang, Ying Li, Jilun Li

**Affiliations:** 1 State Key Laboratories for Agro-biotechnology, China Agricultural University, Beijing, 100193 P.R. China; 2 Institute of Deep-sea Science and Engineering, China Academy of Sciences, Sanya, 572000 P.R. China; 3 Beijing Key Laboratory of Traditional Chinese Medicine Basic Research on Prevention and Treatment for Major Diseases, Experimental Research Center, China Academy of Chinese Medical Sciences, Beijing, 100700 P.R. China; 4 Tianjin Biochip Corporation, Tianjin, 300457 P.R. China; 5 France-China Bio-mineralization and Nano-structure Laboratory, Beijing, 100193 P.R. China

## Abstract

Magnetosome formation by *Magnetospirillum gryphiswaldense* MSR-1 is dependent on iron and oxygen levels. We used transcriptome to evaluate transcriptional profiles of magnetic and non-magnetic MSR-1 cells cultured under high-iron and low-iron conditions. A total of 80 differentially expressed genes (DEGs) were identified, including 53 upregulated and 27 downregulated under high-iron condition. These DEGs belonged to the functional categories of biological regulation, oxidation-reduction process, and ion binding and transport, and were involved in sulfur metabolism and cysteine/methionine metabolism. Comparison with our previous results from transcriptome data under oxygen-controlled conditions indicated that transcription of *mam* or *mms* was not regulated by oxygen or iron signals. 17 common DEGs in iron- and oxygen-transcriptomes were involved in energy production, iron transport, and iron metabolism. Some unknown-function DEGs participate in iron transport and metabolism, and some are potential biomarkers for identification of *Magnetospirillum* strains. IrrA and IrrB regulate iron transport in response to low-oxygen and high-iron signals, respectively. Six transcription factors were predicted to regulate DEGs. Fur and Crp particularly co-regulate DEGs in response to changes in iron or oxygen levels, in a proposed joint regulatory network of DEGs. Our findings provide new insights into biomineralization processes under high- vs. low-iron conditions in magnetotactic bacteria.

## Introduction

Magnetotactic bacteria are a diverse group characterized by the ability to orient themselves and navigate along geomagnetic field lines. They are able to efficiently find low-oxygen environments in fresh water by reference to the earth’s magnetic field. This navigational ability depends on the magnetosome, a specialized organelle consisting of a lipid bilayer membrane surrounding a crystal of the magnetic mineral magnetite (Fe_3_O_4_)^1^. Biosynthesis of magnetosomes by *Magnetospirillum gryphiswaldense* MSR-1, a member of the alpha subclass of Proteobacteria^2^, requires a high concentration of iron ion and a microaerobic (relative dissolved oxygen in 0.5–1.0%) environment^3, 4^. The detailed mechanism of magnetosome formation has been gradually elucidated during the past two decades through a combination of genetic, cell biological, and physiological analyses and several advanced microscopic techniques. The series of steps involved in the process include magnetosome vesicle formation by invagination of inner cell membrane^5, 6^, recruitment of magnetosome membrane proteins, alignment of magnetosome vesicles into a chain^7, 8^, transport of iron into the vesicles^9^, and biomineralization of magnetite crystals. The majority of the genes required for biomineralization are located within a large unstable genomic region termed the magnetosome island (MAI), and belong to four conserved clusters: the *mamAB*, *mamXY*, *mamGFDC*, and *mms6* operons^1, 5, 10^. The *mamAB* cluster encodes factors that are important and sufficient for magnetite biomineralization in MSR-1^11^. Transfer of four gene clusters and *feoAB1* led to construction of a recombinant *Rhodospirillum* that synthesized magnetosomes^12^.

Certain genes located outside the MAI also play key roles in magnetosome formation, particularly during the biomineralization process. Ferric uptake regulator (Fur) protein is a global regulator of iron and oxygen metabolism. In MSR-1, Fur (gene code MGMSRv2_3137) directly regulates expression of ferrous transport system-related genes (*feoAB1*, *feoAB2*), catalase gene *katG*, and superoxide dismutase gene *sodB*. Deletion of *fur* results in fewer and smaller magnetosomes^13, 14^. Iron response regulator protein IrrB (MGMSRv2_3149) helps control iron/oxygen balance, oxidative stress tolerance, and magnetosome formation^15^. Several proteins that participate in general iron metabolism are involved in magnetosome formation, including FeoB1 and FeoB2 (deletion of which results in fewer magnetosomes)^16, 17^, and FeR5 (thioredoxin reductase) and FeR6 (flavin reductase), two bifunctional enzymes that have ferric reduction function and play complementary roles in the process^18^. Li *et al*. demonstrated that magnetite biomineralization also requires periplasmic nitrate reductase (Nap) and nitrite reductase (NirS), both of which are components of the denitrification pathway^19, 20^. Clearly, magnetosome formation is a highly complicated process requiring integration of many metabolic pathways, and genes other than those located within the MAI are involved. The number of genes involved in the process, and their functional relationships, remain unclear, presenting a major obstacle to elucidation of the mechanism of magnetosome formation. We hope to overcome this obstacle using high-throughput mRNA sequencing (RNA-seq) for analysis of the genes.

Through a long series of studies and experimental approaches, we have essentially mastered “the rules” for culturing MSR-1 cells to achieve high-efficiency growth and magnetosome synthesis. Through modification of a single variable (iron or oxygen), we can cause cells to synthesize magnetosomes with high efficiency, or not at all, and that differential transcription of key iron and oxygen metabolism genes tested by RT-qPCR^21^ also ensured the feasibility of transcriptome. Our 2016 report presented transcriptome data from MSR-1 cells under high-oxygen and low-oxygen (oxygen-controlled) conditions, and described cell physiological characteristics that respond to changes in oxygen levels^22^.

In the present study, we evaluated the effects of iron concentration on metabolism and magnetosome formation of MSR-1 cells, in comparison with transcriptome data under high-iron and low-iron conditions. We found that 80 differentially expressed genes (DEGs) in magnetic and non-magnetic cells under the two conditions could be divided into two clusters: 53 upregulated genes and 27 downregulated genes under high-iron condition. Analysis of the transcriptome data indicated that certain genes in multi-metabolic pathways are involved in magnetosome formation, and led to generation of a proposed regulatory network of DEGs. In comparison with transcriptome data under oxygen-controlled conditions, there were both similarities and differences in results from formation of mature magnetosomes. Our findings provide new insights into physiological differences in biomineralization processes under high- vs. low-iron condition.

## Results and Discussion

### Characteristics of magnetic and non-magnetic MSR-1 cells

**Figure 1:**
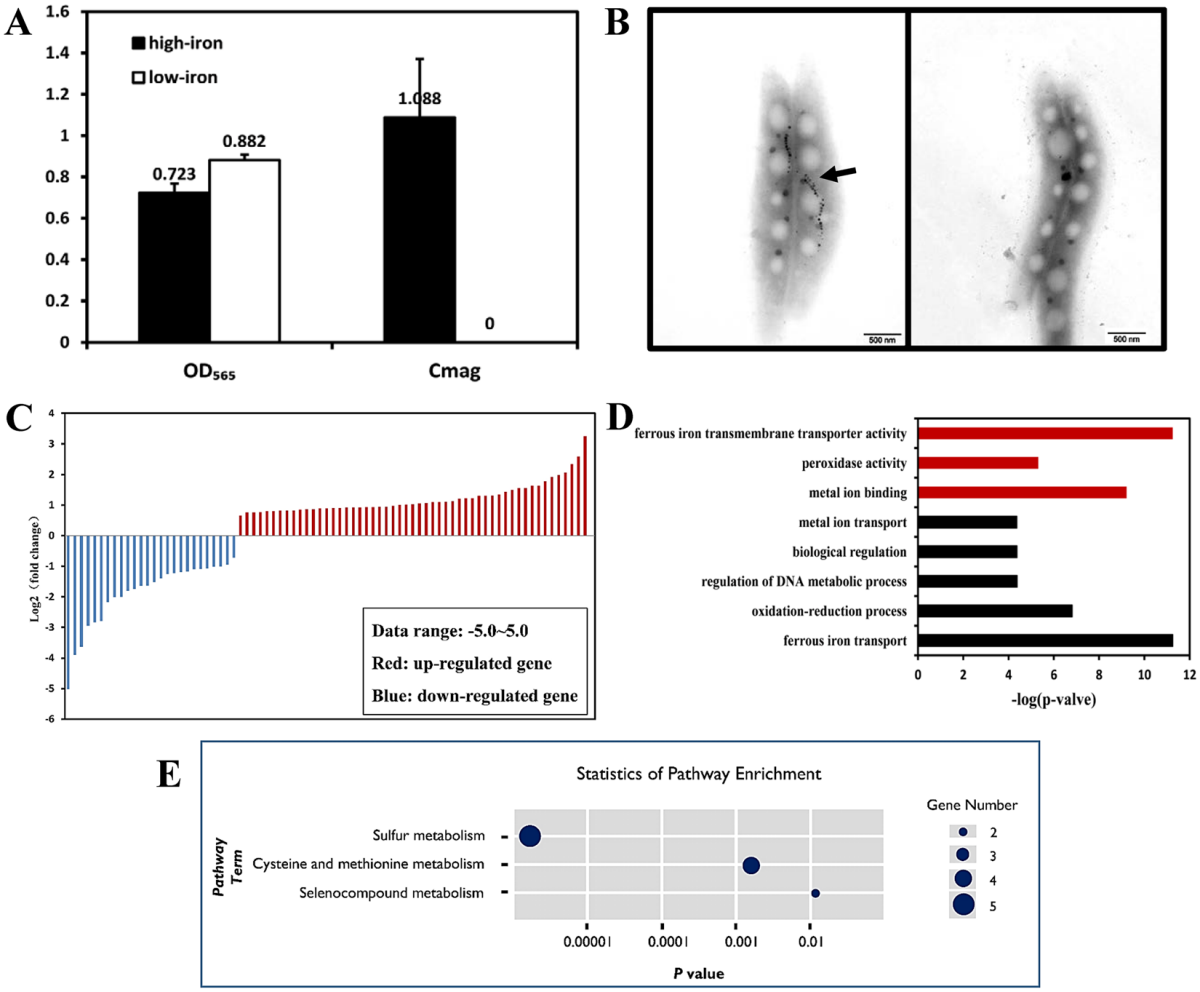
MSR-1 cells cultured under high-iron (20 μM ferric citrate) and low-iron (trace amount iron) conditions, and significant differentially expressed genes (DEGs) under these two conditions. (**A**) Cell growth and magnetic response (Cmag). Cell growth was similar, but Cmag was zero for low-iron cells. (**B**) TEM observations at 18 h. High-iron cells (left) had mature magnetosomes, while low-iron cells (right) did not. Scale bar: 500 nm. (**C**) Heat map illustrating the 80 DEGs: 53 upregulated (red) and 27 downregulated (blue). (**D**) DEGs were assigned GO classification to two categories: biological process (black) and molecular function (red). (**E**) KEGG enrichment analysis showing three pathways with the highest degrees of enrichment (p value < 0.05). Dark-blue circle: number of DEGs in the pathway.

*M. gryphiswaldense* MSR-1 cells were cultured in shaking flasks with and without addition of 20 μM ferric citrate (the two conditions are hereafter termed “high-iron” and “low-iron” cells, respectively). Cell growth was similar under the two conditions; however, magnetic response (Cmag) was zero only for low-iron cells; *i.e*., high-iron cells were magnetic whereas low-iron cells were non-magnetic^21^. After 18 h growth, OD_565_ values were 0.723 for high-iron cells and 0.882 for low-iron cells, Cmag of high-iron cells reached its maximal value (1.088) (Fig. 1A), magnetosomes were mature, and biosynthesis of magnetosomes in high-iron cells was confirmed by TEM observation (Fig. 1B). High- and low-iron cells grown for 18 h were subjected to further experiments as described below.

### Transcriptomic analysis

To evaluate the genes involved in magnetosome formation, with iron considered as the single variable, selected high- and low-iron MSR-1 cells were subjected to transcriptomic analysis. Of 4,258 genes annotated in the SCF1 genome, 3,862 transcripts were detected validly by RNA-seq. The number of mapped cDNA reads was 9.8 × 10^6^ for high-iron cells and 10.4 × 10^6^ for low-iron cells, totaling 4.06 Gb of sequenced MSR-1 cDNA. Nearly all (~99%) of the transcripts were assignable to the genome, illustrating the suitability of RNA-seq for MSR-1 transcriptomic studies.

Differential expression analysis of RNA-seq data using the Cuffdiff software program^23^ revealed 80 genes with significant differential expression. Under high-iron condition, 53 of these DEGs were upregulated and 27 (approximately half as many) were downregulated (Fig. 1C). One gene (MGMSRv2_3980) that encodes a conserved protein of unknown function was transcribed only under low-iron condition (Table S1).

On the basis of Gene Ontology (GO)^24^, DEGs were classified into two categories: biological process and molecular function. The GOseq software package was used to analyze GO-term enrichment^25^. Most of the 80 DEGs observed for high-iron cells participated in oxidation-reduction processes, ferrous iron transport, regulation of DNA metabolic processes, and biological regulation (Fig. 1D; Table S2). In the biological regulation term, five upregulated genes included signal transduction histidine kinases and methyl chemotactic protein genes; eight downregulated genes included iron response regulator gene *irrB*, Nif-specific regulatory genes *nifA* and *glnB* (which encodes nitrogen regulatory gene PII, related to nitrogen fixation regulation), bacterioferritin gene for iron storage and detoxification, and genes related to DNA replication and nucleic acid metabolism. In the oxidation-reduction process term, six upregulated genes included ferredoxin genes, hydrogenase genes, oxidase genes with FAD/NAD(P)-binding domain, and sulfite reductase genes; two downregulated genes included aldehyde dehydrogenase gene and peroxidase gene. In contrast to non-magnetic bacteria, MSR-1 can withstand high concentrations of ferrous iron ion to form magnetosomes, and is not damaged by reactive oxygen species. MSR-1 has therefore developed mechanisms to maintain oxidation pressure in cells. For example, hemerythrin family protein (encoded by MGMSRv2_2221) is responsible for oxygen transport, and binds the O_2_ molecule to a pair of iron atoms (Fe-O-O-Fe)^26^. Upregulation of MGMSRv2_2221 may also have an antioxidative effect. In the molecular function term, seven DEGs (three upregulated, four downregulated) were related to metal ion binding (Fig. 1D; Table S2). Among these, *feoB1* and *feoB2* are responsible for ferrous iron ion transmembrane transport, and their downregulation may be caused by Fur (encoded by MGMSRv2_3137) in MSR-1^13, 27^.

KEGG (Kyoto Encyclopedia of Genes and Genomes) provides a reference database for linking genomic or transcriptomic contents of genes to KEGG reference pathways and thereby inferring systemic behaviors of cells^28^. Three pathways with high KEGG enrichment are shown in Fig. 1E, and Table S3 presents information on the most enriched pathway terms. Sulfur metabolism showed the highest degree of enrichment. Five of the 80 DEGs (MGMSRv2_0470, MGMSRv2_2887, MGMSRv2_1712, MGMSRv2_0469, MGMSRv2_0468) belonged to sulfur metabolism pathway, and each was upregulated under high-iron condition. These genes encode enzymes that participate in multiple steps of sulfate reduction.

The pathway with the second highest degree of enrichment, cysteine and methionine metabolism, included four DEGs: MGMSRv2_2887, MGMSRv2_2672, MGMSRv2_1712, MGMSRv2_2836. MGMSRv2_2887 and MGMSRv2_1712 both encode cysteine synthetase A, which catalyzes conversion of O-acetyl-L-serine to L-cysteine, and both contribute to sulfur metabolism, and cysteine and methionine metabolism. Besides normal physiological metabolism in MSR-1 cells, sulfur metabolism may directly supply elemental S for iron-sulfur (Fe-S) cluster biosynthesis. This complex process includes sulfur production from L-cysteine, iron and sulfur to form a cluster in a scaffold protein, and delivery of the cluster by a carrier to the terminal apotarget^29, 30^. Comparison by RNA-seq of transcription levels of whole genes under high- vs. low-iron conditions suggests that upregulation of enzymes related to sulfur and cysteine metabolism and ferredoxins (both 2Fe-2S and 4Fe-4S) tends to reduce partial oxidation pressure through biosynthesis of Fe-S clusters, which may also participate in diverse biological processes such as respiration, central metabolism, DNA repair, and gene regulation^29^.

In our recent (2016) study of two types of magnetic and non-magnetic MSR-1 cells harvested in a 7.5-L autofermentor using dO_2_ values of 30% (high-oxygen; aerobic) and 0.5% (low-oxygen; microaerobic), we also analyzed transcriptomes under these two conditions^22^. Although the culture conditions differed from those in flask culture (control of iron concentration), two magnetic and non-magnetic cell populations under the high- and low-oxygen conditions were in stationary growth phase and able to produce mature magnetosomes. We evaluated similarities and differences of the two sets of transcriptome data, hereafter referred to as “iron-transcriptome” and “oxygen-transcriptome” data.

### Differential expression of genes encoding Fur family proteins

**Figure 2:**
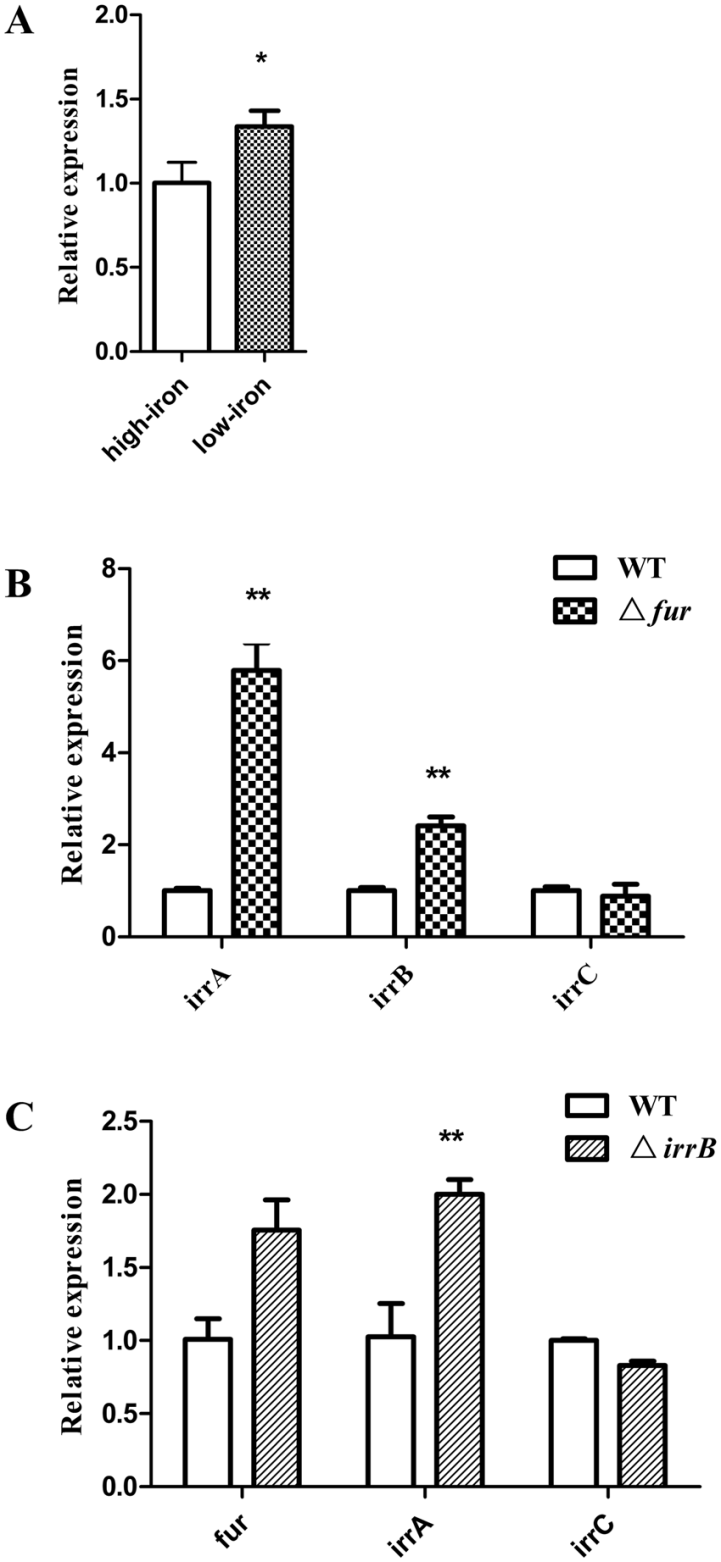
Expression differences (by RT-qPCR) of MSR-1 genes encoding Fur and Fur-like proteins. (**A**) Transcription levels of *irrB* under high-iron and low-iron conditions at 18 h. (**B**) Transcription levels of *irrA*, *irrB*, and *irrC* in WT and Δ*fur* under high-iron condition. (**C**) Transcription levels of *fur*, *irrA*, and *irrC* in WT and Δ*irrB* under high-iron condition. Data are presented as mean ± SD. Means were compared by Student’s t-test, p < 0.05.

The five candidate proteins in MSR-1 responsible for iron and oxygen regulation and belonging to the Fur family are Fur (gene code MGMSRv2_3137), IrrA (MGMSRv2_1721), IrrB (MGMSRv2_3149), IrrC (MGMSRv2_3660), and Zur (zinc uptake regulator; MGMSRv2_2136). In the iron-transcriptome data, only *irrB* showed notable (~2-fold) downregulation under high-iron condition (Table 1). RT-qPCR confirmed that *irrB* transcription was notably downregulated under high-iron condition in stationary phase (Fig. 2A). IrrB level is stable under low-iron condition, whereas under high-iron condition heme initiates degradation of IrrB, leading to expression of IrrB-controlled iron-responsive genes^31^. Under high-iron condition, reduced IrrB expression leads to synthesis of heme proteins such as catalases and peroxidases, and IrrB degradation may be related to oxidative stress^32^. In the oxygen-transcriptome data, only transcription of *irrA* was notably upregulated (4.2-fold) under microaerobic condition (Table 1)^22^. Cmag reached its maximal value and iron storage in cells reached saturation under this condition, thus initiating regulation of IrrA for oxidative balance. Under high-iron condition, transcription levels of *irrA* and *irrB* in Δ*fur* strain (*fu*r-defective mutant) were respectively 5.8- and 2.4-fold higher (p < 0.05) (Fig. 2B), and those of *irrA* and *fur* in Δ*irrB* strain (*irrB*-defective mutant) were both 2-fold higher (Fig. 2C), than in WT. Findings from transcriptomic analysis and RT-qPCR suggest that IrrB is degraded to regulate heme biosynthesis, iron uptake, and iron storage in response to high-iron condition, whereas IrrA regulates iron transport in response to an oxygen signal. Accordingly, Fur and Fur-like proteins in MSR-1 display division of labor and cooperativity in response to varying iron and oxygen conditions.

**Table 1:** Expression differences of *fur* and *fur-like* genes in MSR-1.

Gene name	Gene location	Log_2_ (fold change)^*^	
		high iron/low iron	high oxygen/low oxygen
*fur*	MGMSRv2_3137	0.029	0.689
*irrA*	MGMSRv2_1721	−0.910	2.087^**^
*irrB*	MGMSRv2_3149	1.074^**^	−0.639
*irrC*	MGMSRv2_3660	−0.081	1.368
*zur*	MGMSRv2_2136	−0.499	−0.232

*log_2_ transformation of expression fold change between high- vs. low-iron condition or high- vs. low-oxygen condition.

**p < 0.05.

### Unknown-function proteins involved in substance transport and iron metabolism pathways, and “hidden” information in conserved DEGs

**Figure 3:**
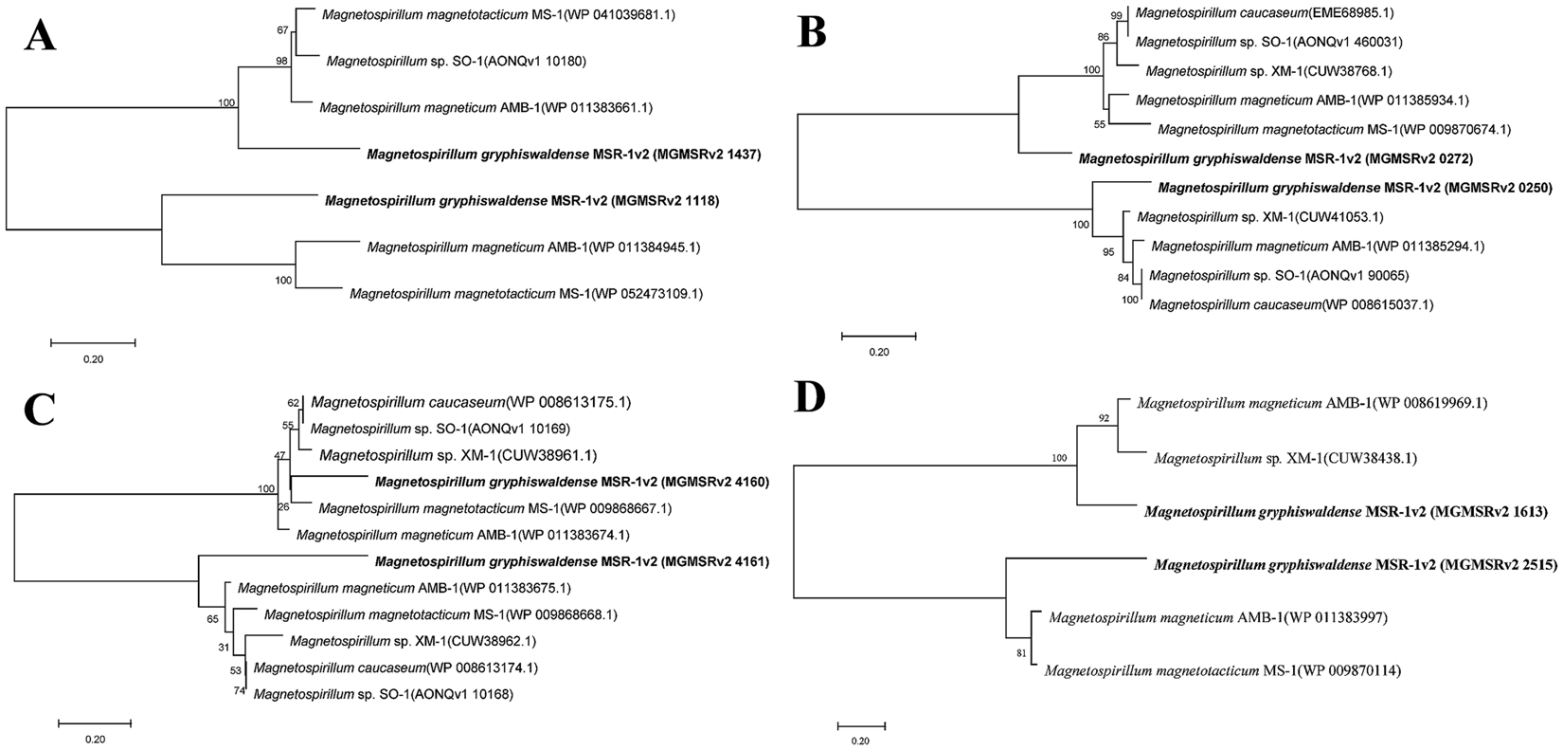
Phylogenetic trees based on sequences of 8 unknown proteins, illustrating relationships within the genus *Magnetospirillum*. Trees were reconstructed by the neighbor-joining method using the MEGA7 software program, and rooted using *M. gryphiswaldense* MSR-1 as outgroup. Numbers at nodes represent bootstrap values, based on 1000 re-samplings. GenBank accession numbers for the unknown protein sequences are shown in parentheses.

Analysis of iron- and oxygen-transcriptome data indicated the presence in MSR-1 of many unknown-function genes related to biomineralization. In the iron-transcriptome, under high-iron condition, transcription of 13 unknown DEGs was upregulated and that of 9 unknown DEGs was downregulated, and transcription of MGMSRv2_1437 and MGMSRv2_2220 was upregulated 4.2- and 6.0-fold, respectively. MGMSRv2_1437 encodes a membrane fusion protein having an RND (resistance–nodulation–cell division) efflux pump domain, and its product may be involved in transport activity regulated by iron signals. BlastP analysis showed 54%, 53% and 56% sequence identity of this MSR-1 protein with homologous proteins in *M. magneticum* AMB-1, *M. magnetotacticum* MS-1, and *Magnetospirillum* sp. SO-1, respectively (Fig. 3A). No such corresponding proteins were found for the protein encoded by MGMSRv2_2220. In the oxygen-transcriptome, under microaerobic condition, transcription of 22 unknown DEGs was upregulated and that of 16 unknown DEGs was downregulated. Of the 22 upregulated DEGs, 13 showed significant (>4.0-fold) upregulation. BlastP analysis showed >50% sequence identity of the proteins encoded by MGMSRv2_0250 (transmembrane protein, transport activity), MGMSRv2_0272, MGMSRv2_1118, MGMSRv2_4160 (transmembrane protein), and MGMSRv2_4161 with homologous proteins in *Magnetospirillum* (AMB-1, XM-1, SO-1, MS-1, *M. caucaseum*) (Fig. 3A–C). The protein encoded by MGMSRv2_4161 belongs to the FeS assembly SUF (sulfur mobilization) system, and that encoded by MGMSRv2_0272 appears to be involved, with its neighbors, in cationic (*e.g*., iron) transport.

Comparison of the iron- and oxygen-transcriptome datasets revealed five unknown-function DEGs in common: MGMSRv2_1306, MGMSRv2_1613, MGMSRv2_2515, MGMSRv2_2779, and MGMSRv2_4010. Transcription of the former three was upregulated under magnetosome-forming conditions (Table 2). The MGMSRv2_1613 product showed 66% sequence identity with the homologous protein in MSR-1, AMB-1, and XM-1 (Fig. 3D), and is predicted to have TadE/G-like domains and to be a putative Flp pilus-assembly protein. MGMSRv2_2515 encoded a cytoplasmic protein with unknown function and insufficient reference information; the protein showed 69% sequence identity with homologues in MSR-1, AMB-1, and MS-1 (Fig. 3D). The protein encoded by MGMSRv2_1306 has a cyclic nucleotide-binding domain; no homologous protein was found in the other strains.

**Table 2:** Transcription of common unknown-function DEGs in iron-controlled and oxygen-controlled transcriptome data.

Gene location	FPKM value				Conserved domain
	high iron	low iron	high oxygen	low oxygen	
MGMSRv2_1306	70.3	17.8	76.0	222.5	Cyclic nucleotide-binding domain
MGMSRv2_1613	62.6	30.2	19.0	58.6	TadE/G-like
MGMSRv2_2515	549.3	202.8	172.2	576.4	—
MGMSRv2_2779	129.1	289.5	101.5	30.1	—
MGMSRv2_4010	36.9	166.7	841.8	161.7	Cystathionine beta-synthase (CBS)

Phylogenetic analysis indicated that the unknown-function proteins described above are conserved in *Magnetospirillum*. Homologous proteins have not been found in other magnetotactic or non-magnetic bacteria. These proteins are likely to be useful markers that will help elucidate *Magnetospirillum* evolution. In transcriptome data, the unknown-function DEGs showed significant upregulation under magnetosome-forming conditions, but more information is needed. We expect that analysis of these conserved unknown-function DEGs, particularly those that show upregulation, will help reveal currently “hidden” information regarding novel substance transport and iron metabolism pathways involved in biomineralization. Our ongoing studies are focused on elucidating the functions of these DEGs.

### Fur and Crp co-regulate DEGs that respond to changes in iron or oxygen concentration

In our 2016 study, seven homologous candidate transcription factors (TFs) were predicted in the oxygen-transcriptome^22^. To assess the regulatory of some TFs between DEGs and high/low iron supply, we predicted potential regulatory elements of DEGs. Analysis of DNA sequences −350 bp upstream of each DEG by the Virtual Footprint program led to prediction of six homologous TFs. The genes and related TFs were integrated into a proposed regulatory network (Fig. S1). Information on TFs and numbers of regulated DEGs is summarized in Table S4. The six candidate TFs were Fur (gene code MGMSRv2_3137), anti-activator for CytR-CRP nucleoside utilization regulon CytR (MGMSRv2_1275), cAMP-activated global TF Crp (MGMSRv2_1601), transcriptional regulator NarL (MGMSRv2_0839), RNA polymerase sigma-H factor SigH (MGMSRv2_3016), and transcriptional activator protein GerE (MGMSRv2_4022). Each of these TFs regulates multiple DEGs, and specific DEGs are regulated by more than one TF.

**Figure 4:**
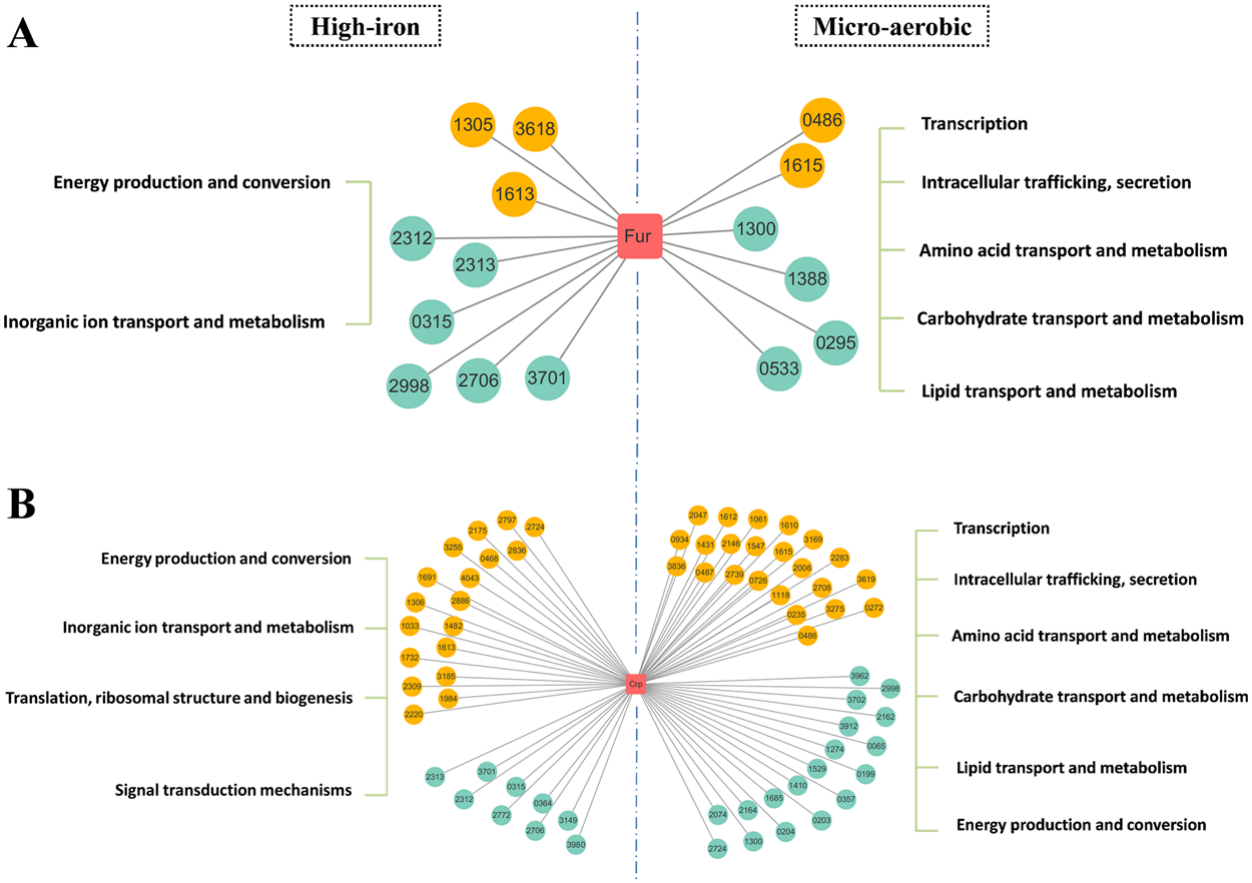
Comparison of proposed regulatory networks for Fur and Crp under high-iron and low-oxygen (microaerobic) conditions. Networks were drawn using the Cytoscape 3 software program. Fur (**A**) and Crp (**B**) are represented by pink rectangles, and DEGs by colored ellipses: orange for upregulated genes, blue for downregulated genes under high-iron condition (**A** and **B**, left) and low-oxygen condition (**A** and **B**, right). COG classifications of DEGs are shown on either side. Four-digit numbers = abbreviated gene IDs; *e.g*., 2724 = MGMSRv2_2724. Black lines: TF regulation of genes.

In the iron-transcriptome under high-iron condition, there were three upregulated genes and five downregulated genes, which included *feoAB1* and *feoB2*, controlled by Fur (Fig. 4A, left). We previously confirmed the DNA-binding ability of upstream *feo* operon by chromatin immunoprecipitation (ChIP), and the sequence of Fur-binding site by DNase I footprinting^13, 14^. In the oxygen-transcriptome under microaerobic condition, two upregulated DEGs (MGMSRv2_0486, MGMSRv2_1615) were regulated by Fur and function in transcription and intracellular trafficking and secretion, while four downregulated DEGs were regulated by Fur and are involved in material transport and metabolism (Fig. 4A, right). The regulatory network of Fur under iron-controlled conditions was related to ferrous ion transport and energy metabolism, in clear distinction to the regulatory network under oxygen-controlled conditions.

The genes *feoAB1*, *feoB2*, MGMSRv2_1305, and MGMSRv2_2998 were controlled by the products of MGMSRv2_1275 (D-ribose-binding periplasmic protein) and MGMSRv2_1601 (catabolite gene activator), which showed high sequence identity with Crp-CytR in *E. coli* (Fig. 4B, left; Fig. S1). In *E. coli*, the cAMP receptor protein (CRP) in combination with the cytidine regulator (CytR) co-regulate the genes involved in nucleoside catabolism and recycling^33^, and Crp is an important global transcriptional regulator for energy metabolism^34^. In the iron-transcriptome of MSR-1 under high-iron condition, two TFs were respectively predicted to control 27 genes (18 upregulated, 9 downregulated) and 20 genes (11 upregulated, 9 downregulated) (Fig. 4B, right; Fig. S1). These regulated DEGs are involved mainly in energy production, energy conversion, and inorganic ion transport. In the oxygen-transcriptome of MSR-1 under microaerobic condition, Crp upregulates 23 DEGs involved primarily in signal transduction and substance transport, and downregulates 18 DEGs involved in energy production and substance metabolism (amino acids, lipids, carbohydrates)^22^ (Fig. 4B, left). MGMSRv2_2724, a class I methyl-accepting chemotaxis protein (MCP) gene, was specifically involved in control of Crp under high-iron and microaerobic conditions. However, the two regulatory modes differ: Crp is upregulated by MGMSRv2_2724 (triggered by high iron), but downregulated by low oxygen.

TFs other than Fur and Crp are present in the iron-transcriptome. Oxygen regulatory protein (MGMSRv2_0839), the best match for NarL of *Pseudomonas aeruginosa*, regulates 34 genes (26 upregulated, 8 downregulated) which were involved in amino acid transport and metabolism and energy production and conversion under high-iron condition, but in *P. aeruginosa*, NarL activity is related to anaerobic energy metabolism and denitrifying growth^35^. MGMSRv2_3016 in MSR-1 (homologous to *sigH* in *Bacillus subtilis*) was annotated as a sigma D factor of RNA polymerase, and it upregulated 9 genes and downregulated 7 genes under high-iron condition. SigH, a member of the Sigma-70 family, regulates transcription of several genes involved in the transition from exponential growth to stationary phase in *Bacillus subtilis* ^36^. MGMSRv2_4022 in MSR-1 (homologous to *gerE* in *B. subtilis*) was annotated as a transcriptional activator protein that regulates 28 genes (19 upregulated, 9 downregulated) under high-iron condition. Functions of SigH and GerE have not been studied in MSR-1. One possibility is that these two TFs regulate various genes involved in energy production and conversion, inorganic ion transport, and ribosome activity.

### Other similarities between iron- and oxygen-transcriptome: involvement of common DEGs in energy production, substrate conversion, iron transport, and metabolism during the magnetosome maturation process

**Figure 5:**
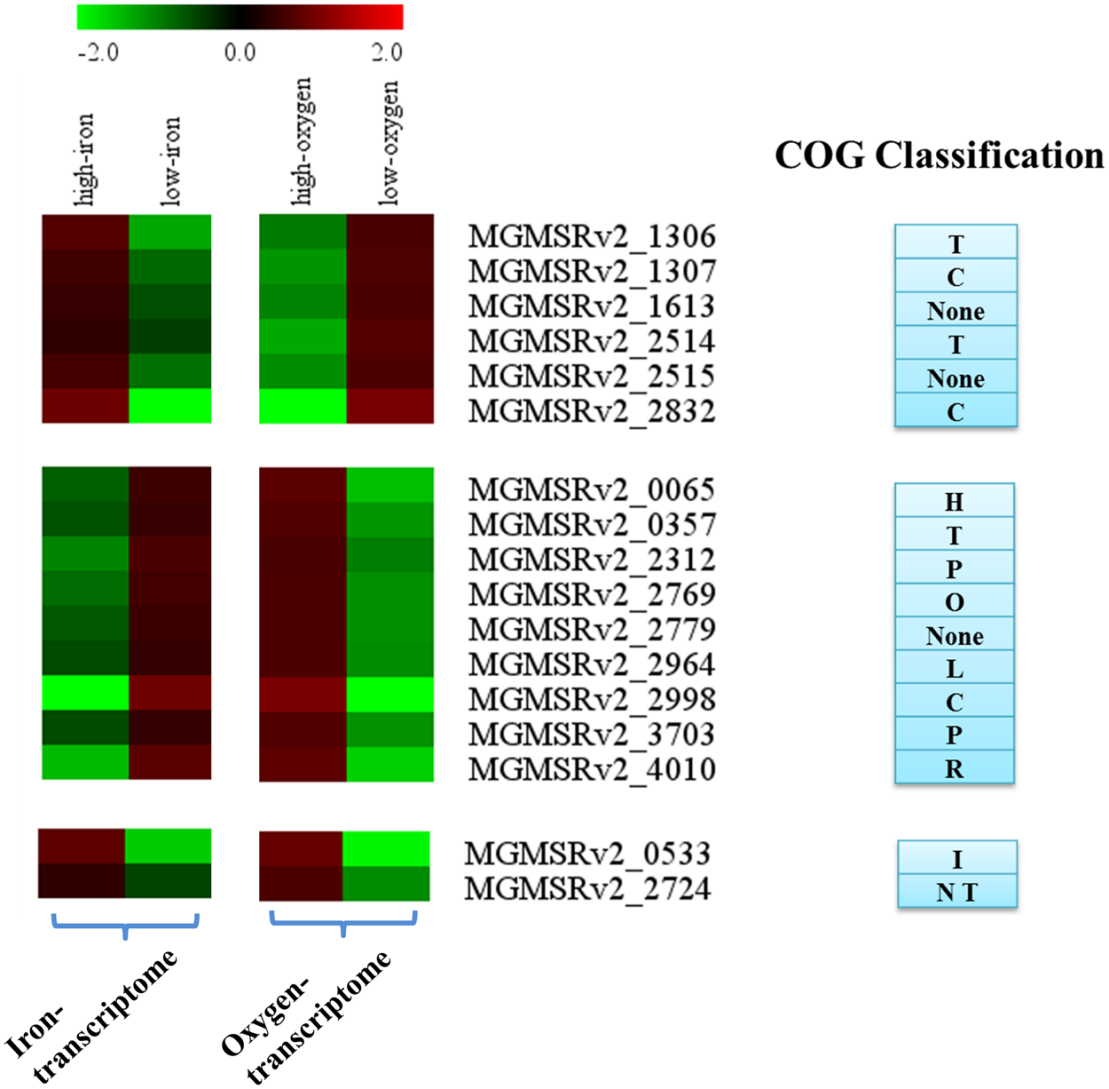
Classification of DEGs from comparison of iron- and oxygen-transcriptome. Hierarchical clustering (HCL) was performed using the Multi Experiment Viewer program, V. 4.8.1. Each expression element was typically the log_2_ transformation of an expression fold change between high- vs. low-iron or high- vs. low-oxygen conditions. 17 DEGs were divided into three groups (left), and corresponding COG designations are shown (right). Descriptions of COG classifications are shown in Table S4.

Comparison of DEGs in iron- and oxygen-transcriptomes revealed 17 common DEGs, which were assigned to three categories on the basis of transcriptional levels (Table S5). The upper group contained six genes (including three with unknown function) that were upregulated under magnetosome-forming conditions (high-iron, low-oxygen) (Fig. 5). Genes in this group participated mainly in energy production and conversion (C) and signal transduction mechanisms (T). The middle group contained nine genes that were downregulated under magnetosome-forming conditions. These genes were classified into 8 COG (cluster of orthologous genes) catalogues (see Fig. 5 and Table S5), and mainly involved in inorganic ion transport and metabolism (P). It is worth attention to downregulated transcription of bacterioferritin *bfr* (MGMSRv2_3703; responsible for iron storage) that may be related to regulation of IrrB^15^ and downregulation of *feoB1* (MGMSRv2_2312) that may be related to regulation of Fur, such that a balance is reached between levels of iron storage and iron uptake when cells have maximal Cmag value and magnetosomes mature gradually. The bottom group contained two genes (MGMSRv2_0533 and MGMSRv2_2724) that were upregulated under high-iron condition but downregulated under microaerobic condition. MGMSRv2_0533 encodes a putative acetyl esterase and is involved in lipid transport and metabolism, while MGMSRv2_2724 encodes a methyl-accepting chemotaxis sensory transducer and is related to chemotaxis.

**Figure 6:**
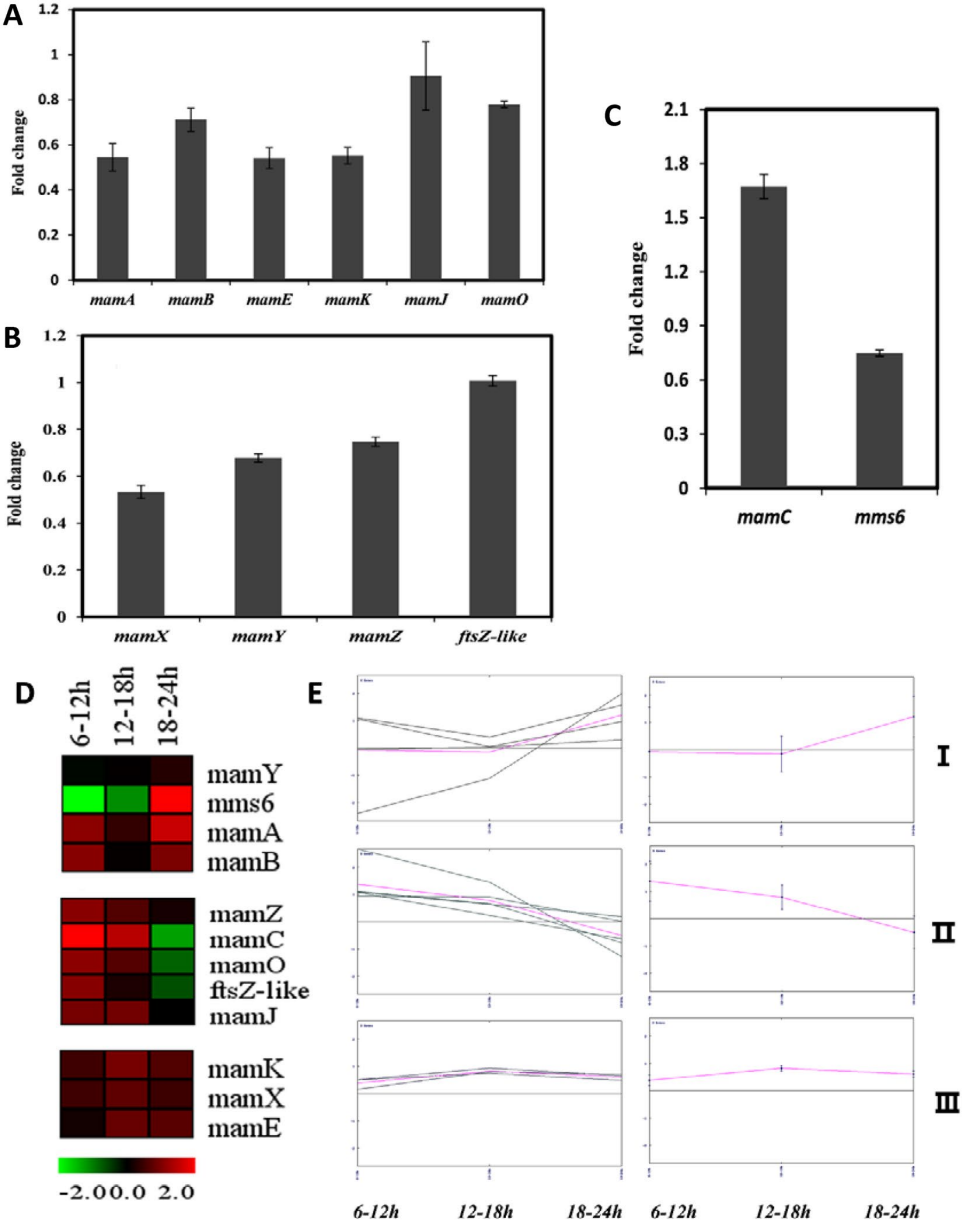
Expression patterns of *mam* and *mms* genes under high- and low-iron conditions. (**A**,**B**,**C**) Fold changes of expression of *mamAB* operon, *mamXY* operon, *mamC*, and *mms6* at 18 h. No clear expression differences for 12 MAI genes were observed under the two conditions at 18 h. (**D**) Representation of “expression rules” from the Multi Experiment Viewer program, V. 4.8.1. The expression matrix is a false-color view on a red-green scale (green = low expression; red = high expression). (**E**) *K*-means clustering shows a consistent trend of expression for members of each cluster. Twelve MAI genes increased at the earlier log phase (6–12 h) of magnetosome formation. The products (proteins) of these genes are located on magnetosome membrane, and may be involved in cell growth and magnetosome formation at the lag phase (0–6 h).

Besides, genes *mam* and *mms*, which encode magnetosome membrane proteins Mam and Mms, showed no significant transcriptional differences between the iron- and oxygen-transcriptomes (Table S6)^22^. Two possible explanations are: (i) the genes are constitutively expressed, and their transcription is not regulated by iron or oxygen signals; (ii) a transcriptional difference between two tested cells appears at some other growth stage (in particular, early stage). Here, 12 MAI genes were selected from the *mms6*, *mamGFDC*, *mamAB*, and *mamXY* operons and analyzed by RT-qPCR. When expression levels of the 12 genes at 18 h under high- vs. low-iron condition were compared, ratios were in the 0.5–2.0 range (Fig. 6A–C). Thus, these genes showed no notable differential expression at 18 h, consistent with results of transcriptome analysis.

To analyze expression patterns of the 12 MAI genes during magnetosome formation, expression levels were evaluated using the Multi Experiment Viewer program, and divided into three distinct clusters (Fig. 6D). Further extraction by *k*-means clustering revealed the expression trend for each gene (Fig. 6E, left) and the overall trend for each cluster (Fig. 6E, right). Cluster I (*mamA*, *mamB*, *mamY*, *mms6*) showed no notable change at log phase (6–18 h) of magnetosome formation, and a stable rate of increase at stationary phase (18–24 h). *mms6*, in contrast to the other three genes, showed decreased expression at log phase. Self-assembly of Mms6 may occur at the initial stage of cell growth, ensuring potential interaction with other Mms proteins^37, 38^. Increased expression of *mms6* at stationary phase is presumably required for upcoming magnetosome formation. Cluster II (*mamJ*, *mamO*, *mamZ*, *ftsZ-like*, *mamC*) showed increased expression from 6–12 h and decrease to a steady state from 12–18 h. Cluster III (*mamK*, *mamE*, *mamX*) showed no notable change of expression at any stage. These findings, taken together, indicate that MAI genes participate in magnetosome membrane biogenesis and magnetite crystal nucleation at 6–12 h, and perhaps even during initial cell growth.

In summary, the studies described here reveal the physiological characteristics of MSR-1 cells that control cell growth under magnetosome-forming conditions, and the proposed regulatory network of TFs and DEGs reflects the coordination and co-dependence of iron and oxygen metabolism. In particular, the TFs Fur and Crp co-regulate many of the DEGs, are components of a novel regulatory relationship for MSR-1, and reflect the important role of global regulators for MSR-1 cells under varying iron and oxygen conditions. In this context, elucidation of currently unknown functions of common DEGs will provide new insights into regulation of magnetosome formation and other physiological processes of magnetic bacteria. Comparative analysis of transcriptosome data under oxygen-controlled conditions suggests that the conserved unknown DEGs in *Magnetospirillum* will be useful biomarkers for identification of strains within this genus, and for better understanding of the biomineralization process.

## Methods

### Growth conditions

*M. gryphiswaldense* MSR-1 cells were grown in sodium lactate medium (SLM) without ferric citrate (SLM (−)) at 30 °C, with 100 rpm shaking, as described previously^16^. In SLM (−), FeSO_4_·7H_2_O was excluded from trace element mixture. Cell cultures (5 mL) were inoculated into SLM (with addition of 0.01 M ferric citrate to final concentration 20 μM) or SLM (−), and grown as above until log stage. The method of MSR-1 cell culture in the flask has been established through multiple repeated pre-experiments^21^, which ensured the stability of samples for transcriptome. Cell growth (estimated by OD_565_) and magnetic response (Cmag) were measured using a modified UV-VIS spectrophotometer (model 2100, UNICO Instrument Co.; Shanghai, China).

### Transmission electron microscopy (TEM)

MSR-1 cells were centrifuged and washed with distilled water, and precipitate was suspended in distilled water. Samples were coated on copper grids, washed twice with distilled water, and observed directly by TEM (model JEM-1230, JEOL; Tokyo, Japan).

### RNA-Seq library construction, sequencing, and data assessment

Cells were centrifuged (12,000 × *g*) at 4 °C, and pellets were grinded in liquid nitrogen and resuspended in 1 mL Trizol reagent (Tiangen Biotech Co.; Beijing, China) for 5 min. Total cellular RNA was isolated by Trizol method as described previously^21^. cDNA fragments were subjected to end-repair and A-tailing, and tailed cDNA was purified and ligated to Illumina adapters. Products were separated using 2% low-melting-point agarose gels, 200- to 250-bp cDNA was purified using a QIAquick Gel Extraction kit (Qiagen; Germany), and purified cDNA was subjected to PCR and sequenced in the Illumina-Solexa sequencing platform as described previously^39^. Data were assessed using a FastQC quality control tool set with default parameters^39^. Cleared data were mapped to the MSR-1 genome using the Burrows-Wheeler Aligner^40^. Coverage at each gene was calculated, and gene expression levels were quantified by Fragments Per Kilobase of exon per Million fragments mapped (FPKM) method^39^. Differentially expressed genes (DEGs) were identified through processing of the general parameter set using the Cuffdiff software program^41^. Genes with q-value < 0.05 and a difference in FPKM between the two conditions 2-fold or higher were considered to be differentially expressed.

### Quantitative PCR

Unique primers were designed for 100-bp and 250-bp segments from 12 MAI genes, and *fur* and *fur*-like genes. MAI genes and primers are listed in Table S7. Primers of *fur* and *fur*-like genes were as described previously^15^. Quantitative PCR was performed in triplicate using a LightCycler 480 SYBR Green I Master Kit (Roche; Mannheim, Germany) in a LightCycler 480 RT-PCR System according to the manufacturer’s instructions^42^. *rpoC* gene (encodes RNA polymerase subunit β′) was used as internal control and reference. For analysis, relative expression of each gene was calculated by the comparative crossing point (C_P_) method and presented as 2^−∆∆Cp^. To evaluate the trend lines of MAI gene expression, we performed hierarchical clustering (HCL) and *k*-means clustering, and generated a distance tree using the Multi Experiment Viewer program^43^.

### Bioinformatics analysis

DEGs were assigned to Gene Ontology (GO) categories using the GOseq method^25^. Differences with p-value < 0.05 were considered significant. KEGG (www.genome.jp/kegg/) is a database of biological systems that integrates genomic, chemical, and systemic functional information^28^. This database was applied for determination of the major pathway in which each DEG participated. Gene promoters (for each gene, the sequence from position −350 bp to −1 bp was selected for analysis) and corresponding TFs were predicted by the Virtual Footprint program, V. 3.0^44^. A proposed regulatory network of DEGs was generated using the Cytoscape 3 software program^45^.

## Electronic supplementary material

Supplemental figure and tables
