# Supplementary material for: Physiological characteristics of Magnetospirillum gryphiswaldense MSR-1 that control cell growth under high-iron and low-oxygen conditions
Source: Sci Rep. 2017 Jun 5;7:2800. doi: 10.1038/s41598-017-03012-4 (PMC5459824; doi:10.1038/s41598-017-03012-4)
Supplement: Supplementary file 1 — Supplemental figure and tables [file 41598_2017_3012_MOESM1_ESM.pdf]

Physiological characteristics of *Magnetospirillum gryphiswaldense* MSR-1 that control cell growth under high-iron and low-oxygen conditions

Qing Wang[1, 5#], Xu Wang [1, 5#], Weijia Zhang[2, 5], Xianyu Li[3], Yuan Zhou[1], Dan Li[1], Yinjia Wang[4], Jiesheng Tian[1, 5], Wei Jiang[1,5], Ziding Zhang[1], Youliang Peng[1], Lei Wang[4], Ying Li[1, 5*], Jilun Li[1,5]

[1] State Key Laboratories for Agro-biotechnology, China Agricultural University, Beijing, 100193, P. R. China.

[2] Institute of Deep-sea Science and Engineering, China Academy of Sciences, Sanya, 572000, P.R. China

[3] Beijing Key Laboratory of Traditional Chinese Medicine Basic Research on Prevention and Treatment for Major Diseases, Experimental Research Center, China Academy of Chinese Medical Sciences, Beijing, 100700, China

[4] Tianjin Biochip Corporation, Tianjin, 300457, P. R. China.

[5] France-China Bio-mineralization and Nano-structure Laboratory, Beijing, 100193, P. R. China.

# These authors contributed equally to this study.

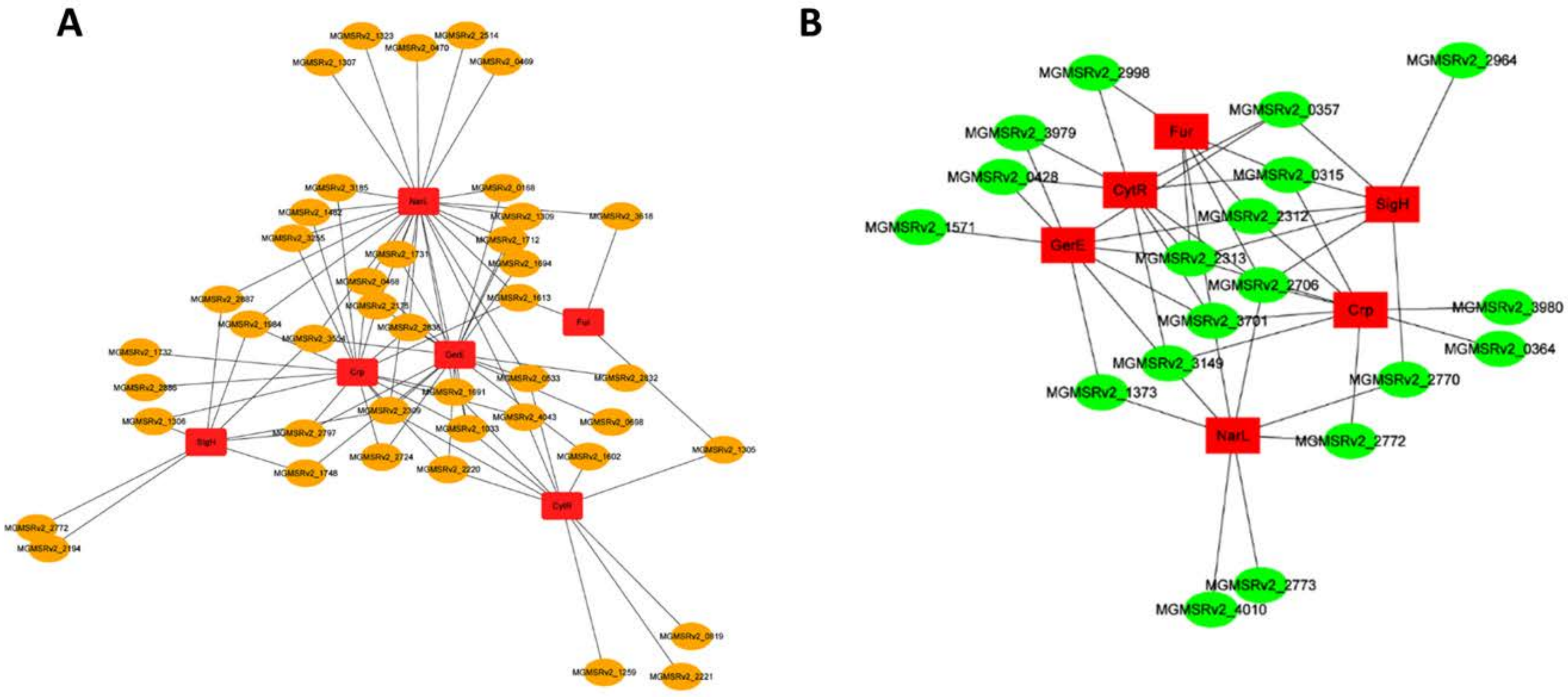

Figure S1. Proposed regulatory network of DEGs using the Cytoscape 3 software program. TFs are represented by red rectangles, and DEGs by colored ellipses: yellow for upregulated genes (A) and green for downregulated genes (B) under high-iron condition. Black lines: TF regulation of genes.

Table S1. Detailed information on DEGs under high and low-iron conditions: gene ID, gene name, and FPKM value used for RNA-seq analysis under the two conditions.

Gene ID	Gene products	FPKM (high-iron)	FPKM (low-iron)	p-value
MGMSRv2_0065	Thiamine biosynthesis protein ThiC (5"-phosphoryl-5-aminoimidazole, 4-amino-5-hydroxymethyl-2-methylpyrimidine-P)	52.6395	125.04	1.01E-06
MGMSRv2_0168	Putative GGDEF: diguanylate cyclase (GGDEF) domain	58.9327	27.5131	0.000102
MGMSRv2_0315	Ferrous iron transport protein B2	23.0569	52.5498	2.1E-05
MGMSRv2_0357	Nif-specific regulatory protein，NifA	16.3435	34.618	0.000879
MGMSRv2_0364	Nitrogen regulatory protein PII,GlnB	28.95	117.393	0.000822
MGMSRv2_0428	Putative AraC-type DNA-binding domain-containing proteins	3.78227	56.4968	2.19E-07
MGMSRv2_0468	Sulfite reductase	112.448	32.6438	1.33E-11
MGMSRv2_0469	Phosphoadenosine phosphosulfate reductase(3'-phosphoadenosine 5'-phosphosulfate sulfotransferase (PAPS reductase)/FAD synthetase and related enzymes)	295.688	119.576	8.93E-07
MGMSRv2_0470	Sulfate adenylyltransferase subunit	236.911	117.479	0.000105

MGMSRv2_0533	Putative acetyl esterase	163.246	32.2799	1.89E-14
MGMSRv2_0698	Conserved protein of unknown function	156.053	89.7898	0.000516
MGMSRv2_0819	Signal transduction histidine-protein kinase BarA (fragment)	71.1842	39.2313	0.000773
MGMSRv2_1033	Ferredoxin--NADP reductase (=Flavodoxin reductase)	1015.7	464.404	3.94E-07
MGMSRv2_1259	Putative sulfide dehydrogenase (flavocytochrome c) flavoprotein chain precursor	258.916	104.431	9.34E-08
MGMSRv2_1305	4Fe-4S ferredoxin, iron-sulfur binding	331.701	134.502	7.9E-08
MGMSRv2_1306	Cyclic nucleotide-binding	70.312	17.7562	6.64E-05
MGMSRv2_1307	Oxidoreductase FAD/NAD(P)-binding	109.376	43.0799	1.43E-05
MGMSRv2_1309	Nickel-dependent hydrogenase, large subunit	119.063	58.4312	0.000116
MGMSRv2_1323	NADH-quinone oxidoreductase chain E (NADH dehydrogenase I, chain E) (NDH-1, chain E)	249.773	131.743	0.000696
MGMSRv2_1337	Conserved hypothetical protein	174.238	91.5624	0.000185
MGMSRv2_1373	Conserved protein of unknown function	58.8058	196.54	2.43E-05
MGMSRv2_1436	Acriflavin resistance protein	36.8293	9.783	2.91E-10
MGMSRv2_1437	Membrane-fusion protein	38.2438	9.15448	1.38E-07

MGMSRv2_1482	3-oxoacyl-[acyl-carrier-protein] synthase II (Beta- ketoacyl-ACP synthase II) (KAS II)	214.926	120.983	0.000774
MGMSRv2_1571	Putative Iron-utilization periplasmic protein	13.2457	102.283	1.47E-12
MGMSRv2_1602	Putative CpaC-related secretion pathway protein	345.669	165.153	0.000221
MGMSRv2_1613	Protein of unknown function	62.607	30.2113	0.000421
MGMSRv2_1691	Methyl-accepting chemotaxis sensory transducer	87.5938	45.3804	0.000569
MGMSRv2_1694	TrkA-C domain protein	205.201	95.1936	3.59E-06
MGMSRv2_1712	Cysteine synthase A, O-acetylserine sulfhydrolase A subunit	68.9543	32.1056	0.000531
MGMSRv2_1731	50S ribosomal subunit protein L13	3895.52	2290.04	0.000151
MGMSRv2_1732	30S ribosomal protein S9	1240.1	667.099	0.000135
MGMSRv2_1748	Conserved protein of unknown function, containing Cytochrome c domain	619.19	320.169	0.000321
MGMSRv2_1811	Putative amino acid dehydrogenase containing NAD(P)-binding domain and ferridoxin-like domain	432.57	256.909	0.000744
MGMSRv2_1984	Protein of unknown function	46789.5	29710.4	0.000819
MGMSRv2_2175	ABC-type branched-chain amino acid transport systems, periplasmic component	191.258	101.694	0.000318

MGMSRv2_2192	Hydrogenase-2 operon protein hybA	183.639	64.9917	3.62E-08
MGMSRv2_2194	Uptake hydrogenase large subunit precursor	172.646	87.5135	6.21E-05
MGMSRv2_2220	Protein of unknown function	90.9857	15.1475	0.000972
MGMSRv2_2221	Putative hemerythrin family protein	156.767	67.2728	0.000413
MGMSRv2_2309	Cell wall protein TIR3	193.054	82.8466	2.71E-05
MGMSRv2_2312	FeoB1	137.097	426.908	1.06E-13
MGMSRv2_2313	FeoA1	486.604	1040.02	0.000109
MGMSRv2_2514	Signal transduction histidine kinase	71.4568	39.1659	0.00085
MGMSRv2_2515	Conserved protein of unknown function	549.26	202.796	2.29E-06
MGMSRv2_2672	Homocysteine-N5-methyltetrahydrofolate transmethylase, B12-dependent	152.841	250.455	0.000716
MGMSRv2_2706	Nodulation protein nolR	20.2423	657.824	0
MGMSRv2_2707	Response regulator receiver domain protein (CheY)	33.5015	232.7	2.22E-16
MGMSRv2_2724	Methyl-accepting chemotaxis sensory transducer	78.2134	41.2363	0.000609
MGMSRv2_2769	Putative bacillithiol peroxidase	127.469	335.863	4.43E-07
MGMSRv2_2770	Conserved membrane protein of unknown function	42.9844	150.784	1.04E-05
MGMSRv2_2771	GTP cyclohydrolase I	96.599	226.383	1.85E-05

MGMSRv2_2772	Conserved hypothetical protein	49.9038	96.6711	0.000554
MGMSRv2_2773	Putative uncharacterized protein	14.5445	45.6646	0.000605
MGMSRv2_2779	Conserved exported protein of unknown function	129.091	289.463	1.94E-05
MGMSRv2_2780	Transcriptional activator ChrR	36.8295	105.719	2.45E-05
MGMSRv2_2796	Alkanesulfonate transporter subunit ; membrane component of ABC superfamily	314.647	169.028	0.000346
MGMSRv2_2797	Alkanesulfonate transporter subunit ; periplasmic-binding component of ABC superfamily	620.189	349.857	0.000252
MGMSRv2_2832	Putative Cytochrome c domain	107.569	11.259	4.71E-10
MGMSRv2_2836	Methylthioribose-1-phosphate isomerase (methionine salvage pathway)	52.7716	22.7884	0.000314
MGMSRv2_2886	Molybdopterin synthase sulfurylase	187.346	93.1194	0.000198
MGMSRv2_2887	Pyridoxal-phosphate (PLP) dependent enzymes family; subunit of cysteine synthase A (O-acetylserine sulfhydrolase A)	264.043	140.468	0.000281
MGMSRv2_2964	Putative Chromosomal replication initiator	216.4	436.203	0.00043
MGMSRv2_2975	Putative Heat shock protein Hsp20	2376.64	1347.82	9.73E-05
MGMSRv2_2998	Aldehyde dehydrogenase B	2.22901	27.6739	1.52E-06

MGMSRv2_3149	Ferric uptake regulation protein	436.529	919.164	1.47E-05
MGMSRv2_3185	Conserved protein of unknown function	44.6195	14.4694	0.000797
MGMSRv2_3255	Methylmalonyl-CoA mutase	156.362	90.157	0.000905
MGMSRv2_3267	Conserved protein of unknown function	134.624	70.451	0.000369
MGMSRv2_3343	Putative transposase IS66 family	28.2899	9.6363	7.82E-05
MGMSRv2_3554	50S ribosomal protein L19	567.27	314.495	0.000785
MGMSRv2_3571	Conserved protein of unknown function	103.651	34.9908	1.62E-07
MGMSRv2_3618	Conserved protein of unknown function	720.419	232.547	0.000153
MGMSRv2_3701	Putative bacterioferritin-associated ferredoxin	64.835	463.702	1.48E-06
MGMSRv2_3703	Bacterioferritin, iron storage and detoxification protein	121.774	244.983	0.000656
MGMSRv2_3827	Cytidylate kinase	294.328	155.519	0.000452
MGMSRv2_3979	Putative cation transport ATPase	4.66052	18.5768	2.6E-05
MGMSRv2_3980	Conserved protein of unknown function	0	61.5886	3.17E-05
MGMSRv2_4010	Conserved protein of unknown function	36.8684	166.686	1.47E-06
MGMSRv2_4043	Elongation factor G (EF-G)	384.862	228.427	0.000449

Table S2. GO enrichment annotation of DEGs, for GO items with p< 0.05. Data shown: GO accession, description, term type, number of genes in each GO item (DEG item), and gene ID.

GO_accession	Description	Term type	p_Value	DEG item	Gene ID
GO:0015684	Ferrous iron transport	Biological process	0.00040989	2	MGMSRv2_2312,MGMSRv2_0315
GO:0055114	Oxidation-reduction process	Biological process	0.008886	8	MGMSRv2_2194,MGMSRv2_1309,MGMSRv2_0468,MGMSRv2_2769,MGMSRv2_2998,MGMSRv2_1323,MGMSRv2_1307,MGMSRv2_1033
GO:0051052	Regulation of DNA metabolic process	Biological process	0.047953	1	MGMSRv2_2964
GO:0065007	Biological regulation	Biological process	0.048199	13	MGMSRv2_2707,MGMSRv2_0428,MGMSRv2_0168,MGMSRv2_3703,MGMSRv2_1691,MGMSRv2_2514,MGMSRv2_0819,MGMSRv2_2964,MGMSRv2_3149,MGMSRv2_0357,MGMSRv2_2724,MGMSRv2_0364,MGMSRv2_2706
GO:0030001	Metal ion transport	Biological process	0.048491	2	MGMSRv2_2312,MGMSRv2_0315

GO:0046872	Metal ion binding	Molecular function	0.001679	7	MGMSRv2_2194,MGMSRv2_1309,MGMSRv2_3703,MGMSRv2_3979,MGMSRv2_2672,MGMSRv2_3255,MGMSRv2_2313
GO:0015093	Ferrous iron transmembrane transporter activity	Molecular function	0.00041	2	MGMSRv2_2312,MGMSRv2_0315
GO:0004601	Peroxidase activity	Molecular function	0.025066	1	MGMSRv2_2769

Table S3. KEGG enrichment of DEGs, and pathway terms showing the greatest degrees of enrichment. Sample number: number of DEGs participating in the homologous terms. Background genes means the number of genes in the metabolism pathway. ID is the number of the KEGG Orthology (KO), and the detail about the metabolism are available in the KO Database.

Term	ID	Sample number	Background number	p-Value	Genes ID
Sulfur metabolism	ko00920	5	11	4.20E-06	MGMSRv2_0470, MGMSRv2_2887, MGMSRv2_0469, MGMSRv2_0468, MGMSRv2_1712
Cysteine and methionine metabolism	ko00270	4	27	0.004654	MGMSRv2_2836,MGMSRv2_2887,MGMSRv2_2672,MGMSRv2_1712
Selenocompound metabolism	ko00450	2	8	0.016779	MGMSRv2_0470,MGMSRv2_2672
Microbial metabolism in diverse environments	ko01120	8	186	0.105627	MGMSRv2_2194,MGMSRv2_3255,MGMSRv2_0470,MGMSRv2_1712, MGMSRv2_2998,MGMSRv2_0469,MGMSRv2_0468,MGMSRv2_2887
Ribosome	ko03010	3	52	0.150526	MGMSRv2_1731,MGMSRv2_1732,MGMSRv2_3554

Glycerolipid metabolism	ko00561	1	7	0.168065	MGMSRv2_2998
Pentose and glucuronate interconversions	ko00040	1	7	0.168065	MGMSRv2_2998
Thiamine metabolism	ko00730	1	8	0.189677	MGMSRv2_0065
Nitrotoluene degradation	ko00633	1	8	0.189677	MGMSRv2_2194
Valine, leucine and isoleucine degradation	ko00280	2	32	0.200559	MGMSRv2_3255,MGMSRv2_2998
Chloroalkane and chloroalkene degradation	ko00625	1	9	0.210735	MGMSRv2_2998
Propanoate metabolism	ko00640	2	35	0.229378	MGMSRv2_3255,MGMSRv2_2998
Limonene and pinene degradation	ko00903	1	12	0.270722	MGMSRv2_2998
One carbon pool by folate	ko00670	1	13	0.289699	MGMSRv2_2672
Folate biosynthesis	ko00790	1	13	0.289699	MGMSRv2_2771
Two-component system	ko02020	5	141	0.301271	MGMSRv2_1691,MGMSRv2_2707,MGMSRv2_2964,MGMSRv2_2724, MGMSRv2_0357

beta-Alanine metabolism	ko00410	1	14	0.308189	MGMSRv2_2998
Histidine metabolism	ko00340	1	15	0.326204	MGMSRv2_2998
Biotin metabolism	ko00780	1	16	0.343756	MGMSRv2_1482
Fatty acid biosynthesis	ko00061	1	16	0.343756	MGMSRv2_1482
Tryptophan metabolism	ko00380	1	16	0.343756	MGMSRv2_2998
Lysine degradation	ko00310	1	17	0.360856	MGMSRv2_2998
Bacterial chemotaxis	ko02030	3	84	0.372317	MGMSRv2_1691,MGMSRv2_2707,MGMSRv2_2724
Fatty acid metabolism	ko00071	1	18	0.377516	MGMSRv2_2998
Glutathione metabolism	ko00480	1	18	0.377516	MGMSRv2_2769
Arginine and proline metabolism	ko00330	1	29	0.534745	MGMSRv2_2998
Glycolysis / Gluconeogenesis	ko00010	1	32	0.57034	MGMSRv2_2998
Glyoxylate and dicarboxylate metabolism	ko00630	1	34	0.592562	MGMSRv2_3255
Carbon fixation pathways	ko00720	1	37	0.623786	MGMSRv2_3255

in prokaryotes					
Metabolic pathways	ko01100	14	571	0.638825	MGMSRv2_1482,MGMSRv2_0065,MGMSRv2_3255,MGMSRv2_2771,MGMSRv2_3827,MGMSRv2_2836,MGMSRv2_2887,MGMSRv2_0470,MGMSRv2_1712,MGMSRv2_2998,MGMSRv2_0469,MGMSRv2_0468,MGMSRv2_1323,MGMSRv2_2672
Pyruvate metabolism	ko00620	1	39	0.643277	MGMSRv2_2998
Pyrimidine metabolism	ko00240	1	40	0.652645	MGMSRv2_3827
Oxidative phosphorylation	ko00190	1	48	0.719349	MGMSRv2_1323
Purine metabolism	ko00230	1	64	0.817114	MGMSRv2_0470
Nitrogen metabolism	ko00910	1	69	0.840101	MGMSRv2_1323
ABC transporters	ko02010	2	125	0.843603	MGMSRv2_1571,MGMSRv2_2175
Biosynthesis of secondary metabolites	ko01110	2	264	0.993884	MGMSRv2_2672,MGMSRv2_2998

Table S4. Transcription factors (TFs) predicted for regulation of DEGs.

**Transcription factor**		**Candidate in MSR-1**			**Number of regulated genes**		**p-Value***
**Name**	**Strain**	**Gene**	**Gene product**	**Identity (%)**	**Up- regulated**	**Down- regulated**	
Fur	*M. gryphiswaldense* MSR-1	MGMSRv2_3137	Ferric uptake regulator	100%	3	6	0.0062
CytR	*E. coli* (strain K12)	MGMSRv2_1275	D-ribose-binding periplasmic protein	25.61	11	9	0.042
Crp	*E. coli* (strain K12)	MGMSRv2_1601	Catabolite gene activator	29.27	18	9	0.029
NarL	*P. aeruginosa* (PAO1)	MGMSRv2_0839	Putative oxygen regulatory protein	34.29	26	8	0.032
SigH	*B. subtilis (strain 168)*	MGMSRv2_3016	Sigma D factor of RNA polymerase	27.27	9	7	0.036
GerE	*B. subtilis* (strain 168)	MGMSRv2_4022	Putative transcriptional activator protein	47.46	19	9	0.017

* p-value <0.05

Table S5. Information on DEGs from comparison of iron-controlled vs. oxygen-controlled transcriptomes, and descriptions of COG classifications.

Gene_ID	Gene products	FPKM (high-oxygen)	FPKM (low-oxygen)	Fold change (high-oxygen/low-oxygen)	FPKM (high-iron)	FPKM (low-iron)	Fold change (high-iron/low-iron)	COG
MGMSRv2_1306	Conserved protein of unknown function, containing cyclic nucleotide-binding-like	76.0485	222.455	0.34186	70.312	17.7562	3.9598563	T
MGMSRv2_1307	Putative dihydroorotate dehydrogenase (electron transfer subunit)	43.9466	152.609	0.287969	109.376	43.0799	2.5389103	C
MGMSRv2_1613	Protein of unknown function	19.0019	58.5595	0.324489	62.607	30.2113	2.0723041	None
MGMSRv2_2514	Signal transduction histidine kinase	37.1615	149.858	0.247978	71.4568	39.1659	1.8244646	T
MGMSRv2_2515	Protein of unknown function	172.191	576.437	0.298716	549.26	202.796	2.7084361	None
MGMSRv2_2832	Putative Cytochrome c domain	6.71356	169.17	0.039685	107.569	11.259	9.5540457	C
MGMSRv2_0065	Thiamine biosynthesis protein ThiC	284.799	60.9792	4.670429	52.6395	125.04	0.4209813	H
MGMSRv2_0357	Nif-specific regulatory protein	185.057	51.9591	3.56159	16.3435	34.618	0.4721099	T
MGMSRv2_2312	FeoB1	179.243	59.8701	2.993865	137.097	426.908	0.3211394	P
MGMSRv2_2769	Putative bacillithiol peroxidase	118.82	35.0817	3.386951	127.469	335.863	0.3795268	O

MGMSRv2_2779	Exported protein of unknown function	101.485	30.0468	3.377564	129.091	289.463	0.4459672	None
MGMSRv2_2964	Putative Chromosomal replication initiator	225.877	68.9921	3.273955	216.4	436.203	0.4960993	L
MGMSRv2_2998	Aldehyde dehydrogenase B	113.377	2.9239	38.77595	2.22901	27.6739	0.0805456	C
MGMSRv2_3703	Bacterioferritin, iron storage and detoxification protein	127.898	37.135	3.444136	121.774	244.983	0.4970712	P
MGMSRv2_4010	Conserved protein of unknown function	841.828	161.666	5.207205	36.8684	166.686	0.2211847	R
MGMSRv2_0533	Putative acetyl esterase	237.963	35.9135	6.626004	163.246	32.2799	5.0572028	I
MGMSRv2_2724	Methyl-accepting chemotaxis sensory transducer	54.9298	16.725	3.284293	78.2134	41.2363	1.8967124	NT

*COG classification: A, RNA processing and modification; B, Chromatin structure and dynamics; C, Energy production and conversion; D, Cell cycle control, cell division, chromosome partitioning; E, Amino acid transport and metabolism; F, Nucleotide transport and metabolism; G, Carbohydrate transport and metabolism; H, Coenzyme transport and metabolism; I, Lipid transport and metabolism; J, Translation, ribosomal structure and biogenesis; K, Transcription; L, Replication, recombination and repair; M, Cell wall/membrane/envelope biogenesis; N, Cell motility; O, Posttranslational modification, protein turnover, chaperones; P, Inorganic ion transport and metabolism; Q, Secondary metabolites biosynthesis, transport and catabolism; R, General function prediction only; S, Function unknown; T, Signal transduction mechanisms; U, Intracellular trafficking, secretion, and vesicular transport; V, Defense mechanisms; W, Extracellular structures; Y, Nuclear structure; Z, Cytoskeleton; None, No COG.

Table S6. *mam* genes showing no significant difference in transcription level.

Gene ID	Gene	FPKM(high-iron)	FPKM(low-iron)	Log2(fold change)	q value	Significant
MGMSRv2_2321	*mamY*	163.961	139.092	-0.237309	0.68	no
MGMSRv2_2322	*mamX*	205.689	190.091	-0.113775	0.86	no
MGMSRv2_2323	*mamZ*	146.976	117.216	-0.326413	0.52	no
MGMSRv2_2324	*ftsz-like*	50.6803	60.9183	0.265451	0.71	no
MGMSRv2_2365	*mamU*	27.0006	35.4416	0.392453	0.67	no
MGMSRv2_2366	*mamT*	154.279	98.1922	-0.651858	0.27	no
MGMSRv2_2367	*mamS*	194.202	196.302	0.0155171	0.98	no
MGMSRv2_2368	*mamB*	490.283	461.045	-0.0887098	0.87	no
MGMSRv2_2369	*mamR*	767.922	554.678	-0.469309	0.42	no
MGMSRv2_2370	*mamQ*	554.827	542.54	-0.0323096	0.96	no
MGMSRv2_2371	*mamA*	640.641	619.297	-0.0488855	0.94	no
MGMSRv2_2372	*mamP*	418.758	332.74	-0.33172	0.51	no
MGMSRv2_2373	*mamO*	416.398	395.043	-0.0759548	0.88	no
MGMSRv2_2374	*mamN*	217.392	208.96	-0.0570714	0.93	no
MGMSRv2_2375	*mamM*	371.099	295.753	-0.327411	0.50872	no
MGMSRv2_2376	*mamL*	518.68	286.652	-0.855545	0.16	no
MGMSRv2_2377	*mamK*	220.051	189.702	-0.214099	0.70	no
MGMSRv2_2378	*mamJ*	240.09	186.516	-0.364277	0.47	no
MGMSRv2_2379	*mamE*	632.474	520.322	-0.2816	0.50	no
MGMSRv2_2380	*mamI*	449.657	544.552	0.276244	0.73	no
MGMSRv2_2381	*mamH*	168.118	138.026	-0.284529	0.60	no
MGMSRv2_2393	*mamC*	133.093	78.1884	-0.767408	0.28	no
MGMSRv2_2394	*mamD*	287.129	182.447	-0.654221	0.15	no
MGMSRv2_2395	*mamF*	735.742	558.147	-0.398554	0.46	no
MGMSRv2_2396	*mms6*	93.6725	47.2774	-0.986476	0.17	no
MGMSRv2_2411	*mamW*	296.288	231.746	-0.354451	0.55	no

(all q value>0.05)

Table S7. Primers used in this study.

Gene name	Location	Prime name	Sequence (5'-3')	Product length (bp)
mamC	MGMSRv2_2393	C-F	CCGCCGCCCTTGCCAAGAAT	165
		C-R	GCTCCCAACGAGACCA	
mamE	MGMSRv2_2379	E-F	ATAGCGTGGTTAGCGTGA	224
		E-R	CCACATCGTCCTTTACGGT	
mamK	MGMSRv2_2377	K-F	TGGTTGGATACCCGAAG	215
		K-R	AAATCTCGTCGTTGGCG	
mamO	MGMSRv2_2373	O-F	TCTTCCTGACCAATGTCGT	213
		O-R	ATTTCCAACACCGCCTTG	
mamA	MGMSRv2_2371	A-F	TGCTTGATGAAGTTACCCTG	178
		A-R	ACCACTGCCTCGGAATAA	
mamB	MGMSRv2_2368	B-F	CTGATGTATGGGTCGGTGGT	177
		B-R	AACGGTTGTCCCAGGCATT	
mamJ	MGMSRv2_2378	J-F	ACCCGCCAGCCTAACAAGAT	189
		J-R	CGACCACATTGCTTCCAAC	
mms6	MGMSRv2_2396	mms6-F	AAGGTTGGCGCGGGCAAGGT	249
		mms6-R	CCCCCAAGCCCCAAGGCCCATA	
mamX,	MGMSRv2_2322	X-F	CGCTGTTCAATGCCAATC	261
		X-R	CAACGCTGCCCTTCTTCA	
mamY	MGMSRv2_2321	Y-F	CCCAGGAAATCACCCAAGA	246
		Y-R	CGAAATAGGCGTTAGTCAGG	
mamZ	MGMSRv2_2324	Z-F	GCCCGGTGCAGGAGATTACT	231
		Z-R	TCTGGCTGTTGGTGGAGGTG	
rpoC	MGMSRv2_0030	rpoC-F	ATCCGTATTTCCATCGCCTCCC	164
		rpoC-R	TTGCCGCACAAGCATTCGT	
